# Detection of Diabetic Retinopathy Using Bichannel Convolutional Neural Network

**DOI:** 10.1155/2020/9139713

**Published:** 2020-06-19

**Authors:** Shu-I Pao, Hong-Zin Lin, Ke-Hung Chien, Ming-Cheng Tai, Jiann-Torng Chen, Gen-Min Lin

**Affiliations:** ^1^Department of Ophthalmology, Tri-Service General Hospital and National Defense Medical Center, Taipei 114, Taiwan; ^2^Department of Ophthalmology, Buddhist Tzu Chi General Hospital, Hualien 970, Taiwan; ^3^Institute of Medical Sciences, Tzu Chi University, Hualien 970, Taiwan; ^4^Department of Medicine, Hualien Armed Forces General Hospital, Hualien 971, Taiwan; ^5^Department of Medicine, Tri-Service General Hospital and National Defense Medical Center, Taipei 114, Taiwan; ^6^Department of Preventive Medicine, Northwestern University, Chicago, IL 60611, USA

## Abstract

Deep learning of fundus photograph has emerged as a practical and cost-effective technique for automatic screening and diagnosis of severer diabetic retinopathy (DR). The entropy image of luminance of fundus photograph has been demonstrated to increase the detection performance for referable DR using a convolutional neural network- (CNN-) based system. In this paper, the entropy image computed by using the green component of fundus photograph is proposed. In addition, image enhancement by unsharp masking (UM) is utilized for preprocessing before calculating the entropy images. The bichannel CNN incorporating the features of both the entropy images of the gray level and the green component preprocessed by UM is also proposed to improve the detection performance of referable DR by deep learning.

## 1. Introduction

Retinopathy often refers to retinal microvascular damage resulted from abnormal blood flow and may cause visual impairment. Frequently, retinopathy is an ocular manifestation of diabetes or hypertension. It is predicted that around 600 million people will have diabetes by 2040, with one-third estimated to have diabetic retinopathy (DR). Early detection of DR by regular clinical examination and prompt treatment are essential for the prevention of vision impairment and to raise living quality [1–4]. Fundus photography has been widely used worldwide to be an ophthalmologic screening tool utilized for detecting DR [5]. Retinal telescreening with remote interpretation by an expert for evaluation of DR may be useful in helping rural and medically underserved patients [6]. However, some diabetic patients cannot afford the cost of an ophthalmologist visit [7]. In addition, the assessment of DR severity needs specialized expertise, and the agreement of interpretation results may vary from the graders [8]. Automated assessment systems for DR image may provide clinically effective and cost-effective detection of retinopathy and therefore help the prevention of diabetic-associated blindness [9].

Artificial intelligence has the potential to revolutionize the traditional diagnosis method for eye disease and bring out a significant clinical impact on promoting ophthalmic health care service [10–13]. Automated DR detections have been previously studied [14–18]. Deep learning of fundus photograph has emerged as a practical technique for automatic screening and diagnosis of DR. The effective deep learning system is able to correctly and automatically identify severer DR with equal or better accuracy than the trained graders and retina specialists [19], and thus, it can benefit the patients in medically underserved areas that have limited numbers of ophthalmologists and rare medical resources. The convolutional neural network (CNN), a core model of deep learning in computer vision, has yielded impressive results in terms of prediction and diagnosis in medical image classification. The entropy image of luminance of fundus photograph, which involves measuring the complexity of a retinal image, has been demonstrated to increase the detection performance of referable DR for a CNN-based system in [18].

Several image processing methods have been discussed for the retinal image to meliorate microaneurysm detection in [20]. An image enhancement method is proposed to increase the contrast and improve the overall appearance of retinal image by taking the information of color models in [21]. The green component of the RGB retinal image is used for preprocessing of improved blood vessel and optic disc segmentation in [22]. In [23], the green component of retinal image is also used to train a network to segment the macular region. In this paper, we extract the green component of color fundus photograph and enhance the details by unsharp masking (UM), a classical tool for sharpness enhancement [24] and has been applied to fundus photograph [25, 26] and medical images [27, 28], before calculating the entropy images. The proposed bichannel CNN is trained by incorporating the features of both the entropy images from the gray level and the green component of fundus photograph preprocessed by UM to heighten the detection of referable DR.

## 2. Materials and Methods

### 2.1. Dataset and Grading

The total of 35,126 color fundus photographs with the sizes from 433 × 289 pixels to 5184 × 3456 pixels is obtained from the publicly available “Kaggle Diabetic Retinopathy” dataset [29–34], which is acquired by using various digital fundus cameras in several eye centers in California and around the United States. We select 21,123 color fundus photographs with good image quality. The experimental setup is the same with that in [18].

The retinal images obtained from the Kaggle dataset have been independently graded by well-trained clinicians according to the International Clinical Diabetic Retinopathy Disease Severity Scale: no apparent retinopathy (grade 0), mild nonproliferative DR (grade 1), moderate nonproliferative DR (grade 2), severe nonproliferative DR (grade 3), and proliferative DR (grade 4) [35]. The image numbers for grade 0, grade 1, grade 2, grade 3, and grade 4 are 16,500, 1,333, 2,000, 645, and 645, respectively. Referable DR is defined as the presence of severe DR grades 2–4 (3,290 images, 15.6%) that requires a referral to the eye specialist and used as the output for the deep learning in this paper.

### 2.2. Data Augmentation

The 21,123 eligible color fundus photographs are resized to a resolution of 100 × 100 pixels. The resized images are increased by data augmentation using flipping and rotation. The retinal images of grade 1–grade 4 are randomly selected from the augmented images to the numbers 4,375 (13.26%), 4,375 (13.26%), 3,875 (11.74%), and 3,875 (11.74%), respectively, for a total of 16,500 images balanced with grade 0 (16500, 50%). In total, 33,000 images are used for experiments. To compose a total of 30,000 retinal images in the training set, 15,000 images of grade 0 and 15,000 images of grade 1–grade 4 (4,000, 4,000, 3,500, and 3,500, respectively) are randomly chosen. Then, the remaining 1,500 images of grade 0 and the 1,500 images of grade 1–grade 4 (375, 375, 375, and 375, respectively) are utilized as the test set.

### 2.3. Preprocessing of Retinal Images

**Figure 1:**
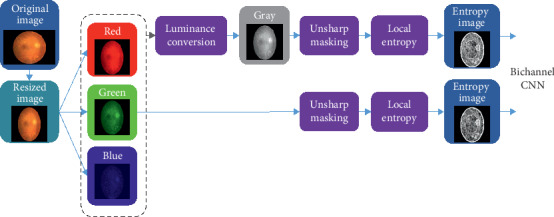
The dataflow of preprocessing for the retinal images.

Figure 1 illustrates the dataflow of preprocessing for the retinal images in the proposed method. After resizing to a resolution of 100 × 100 from original retinal fundus photograph, the green component is extracted from the retinal image with the RGB color model. The luminance conversion is calculated from red (*R*), green (*G*), and blue (*B*) components of color retinal fundus photograph by equation (1) to obtain the gray level image as in [18].

(1) $$\begin{array}{c} \text{Gray Level}=0.299*R+0.587*G+0.114*B. \end{array}$$

In order to enhance of the details of the retinal image, the UM technique is utilized to amplify the high-frequency parts of the gray level (luminance) and the green component of the retinal image before computing the entropy images. An unsharp mask is obtained by subtracting a Gaussian blurred image from the original image. The unsharp mask contains high-frequency information associated with edges. Then, a scaled mask is added to the original image to create an enhanced image.

The entropy image of luminance of fundus photograph represents the complexity of the original retinal image and benefits the training of the CNN-based deep learning system [18]. The values in the entropy image are calculated locally from *n* x *n* blocks to measure the heterogeneity. The entropy is a function of the probability distribution of the local intensity. Equations (2) and (3) represent the values in the entropy images for the two inputs of the proposed bichannel CNN.

(2) $$\begin{array}{c} E_{\text{gray}}=-\underset{i}{\sum }P_{\text{gray}\_\text{UM}}\left(i\right)\times \;\log_{2}P_{\text{gray}\_\text{UM}}\left(i\right), \end{array}$$

(3) $$\begin{array}{c} E_{\text{green}}=-\underset{i}{\sum }P_{\text{green}\_\text{UM}}\left(i\right)\times \;\log_{2}P_{\text{green}\_\text{UM}}\left(i\right), \end{array}$$

where *P*_gray_UM_ (*i*) and *P*_green_UM_ (*i*) denote the relative frequencies associated with the *i*-th intensity within a *n* x *n* block in the gray level and the green component of retinal image, respectively, after processing by UM. Since the result of *n* = 9 reaches the maximal accuracy among the various block sizes as exhibited in [18], accordingly, *n* = 9 is also chosen to calculate the entropy images of the gray level and the green component of retinal image after applying UM in our experiments. The entropy images use the statistical characteristics of the local areas and present the local structural information of the retinal images. The pixels of the entropy image with intensities between 0 and 255 are rescaled to the values between 0 and 1 to be the CNN inputs.

### 2.4. Deep Learning by Bichannel Convolutional Neural Network

**Figure 2:**
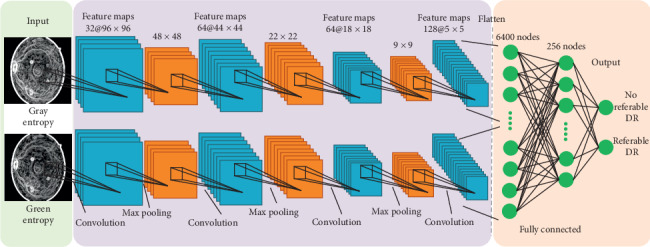
The diagram of the proposed bichannel CNN model.

The convolutional neural network (CNN) is used for the feature learning of referable DR in this study. We construct a bichannel CNN model to simultaneously process the entropy images of luminance (gray level) and the green component after processing by UM as shown in Figure 2. For each channel, 4 convolutional layers are with 5 × 5 kernels, and the numbers of filters are 32, 64, 64, and 128 in successive layers. Maximum pooling, rectified linear unit activation function, and dropout (set to 0.3), to prevent overfitting, are used. After flattening from the two channels, the fully connected layers are linked to statistically determine the detection of referable DR. The proposed referable DR detection method is coded by TensorFlow software with Python. The cross-entropy loss function and the Adam algorithm with learning rate 0.0001 are adopted for training the network.

## 3. Results and Discussion

Performance evaluation consists of several standard measurements including accuracy, sensitivity, specificity, and the area under the receiver-operating characteristic curve (AUC of the ROC curve) of the automatic screening for the presence of referable DR. We use the clinically defined referable DR in the Kaggle dataset as the benchmark to validate the proposed algorithm.

Table 1 compares the detection accuracy, sensitivity, and specificity for referable DR by various retinal image inputs to the CNN. As revealed in [18], the result of the entropy image of the gray level outperforms that of the original photograph. The proposed method utilizing the entropy image of the green component provides better accuracy and sensitivity than the entropy of luminance in the fundus photograph. Employing the entropy image of the green component can improve the accuracy and prevent under diagnosis by elevating sensitivity with a negligible loss of specificity. By applying the bichannel CNN with the two inputs of the entropy images from the gray level and green component, the performance is better than that of the single-channel CNN of individual input.

All of accuracy, sensitivity, and specificity increase when the entropy image is obtained from the preprocessed gray level or green component by UM. Since the contrast is enhanced by UM, the corresponding bichannel CNN yields the best results for deep learning. The measurements of the proposed bichannel CNN model regarding accuracy, sensitivity, and specificity are 87.83%, 77.81%, and 93.88%, respectively, which are better than 86.10%, 73.24%, and 93.81%, respectively, of the previous study [18], which only implements the single-channel CNN by training the entropy image of the gray level of fundus photograph alone.

**Figure 3:**
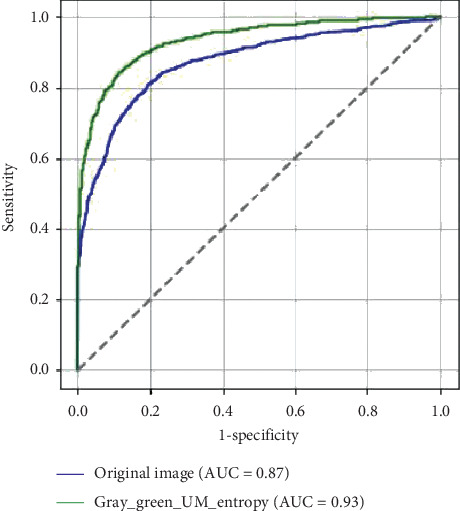
AUC of the ROC curve comparison of deep learning by the proposed method and the original photograph.

Furthermore, the AUC of the ROC curve is used as the integral performance index. As shown in Figure 3, the proposed bichannel CNN method obtains the AUC of 0.93, which is better than 0.87 of the CNN trained by original photograph.

**Figure 4:**
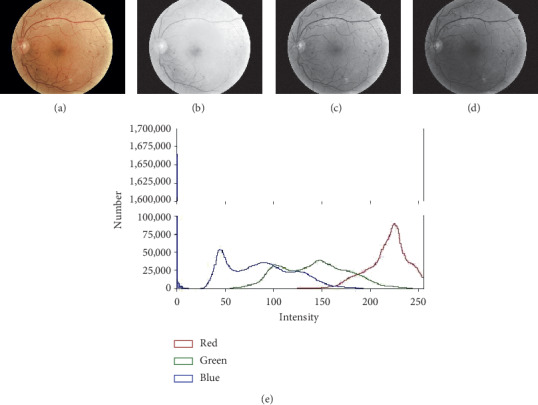
Original retinal photograph (grade 3) and the histograms. (a) Original color (2592 × 1944). (b) Red component. (c) Green component. (d) Blue component. (e) *R*, *G*, and *B* histograms.

Figure 4 shows the color fundus photograph of DR grade 3 with the resolution of 2592 × 1944, the respective intensities of monochromatic *R*, *G*, and *B* components, and the histograms. Excluding the peak values for all of *R*, *G*, and *B* components with very low intensities in the histograms resulting from the dark background, broader distribution of the green component is observed from Figure 4(e). The histogram in Figure 4(e) can depict the amount of contrast, which is the measurement of brightness difference between light and dark regions in the retinal image. Broad histogram expresses the image with significant contrast, whereas narrow histogram represents less contrast.

From the observation of red, green, and blue components for monochromatic fundus photograph in previous studies [36, 37], green light provides the best overall view of the retina and displays excellent contrast because the retinal pigmentations reflect green light more than blue light. Hence, green filter is utilized for enhancing the visibility of retinal vasculature, drusen, hemorrhage, and exudate. This finding motivates us to extract the green component from color fundus photograph before calculating the entropy image for the input of the CNN. It benefits the learning of the CNN to recognize lesions using the local features by entropy images.

**Figure 5:**
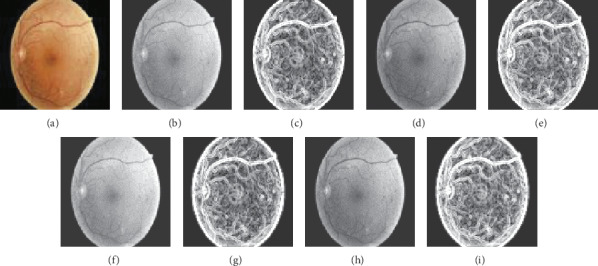
Resized (100 × 100), preprocessed, and entropy images of the retinal image (grade 3). (a) Color. (b) Gray level. (c) Entropy of (b). (d) Green component (e) Entropy of (d). (f) UM of (b). (g) Entropy of (f). (h) UM of (d). (i) Entropy of (h).

Figure 5 illustrates the resized, preprocessed, and entropy images in the dataflow of the proposed system for Figure 4(a). Our proposed approach utilizes UM to increase contrast, which can display a significant visual impact by emphasizing texture in the retinal image. From Figures 5(f) and 5(h), more structural information is enhanced by UM than from Figures 5(b) and 5(d). Severer DR gives rise to higher heterogeneity than mild or no DR in a retinal image. To discriminate the characteristics of no or mild DR and severer DR, the complexities of the gray level and green component images are analyzed by computing local entropy. The low-entropy image has low complexity; instead, the high-entropy image represents high complexity among neighboring pixels. Severer DR images may contain neovascularization or the lesions more than just microaneurysms and thus have more heterogeneous areas with high local entropy values; on the contrary, no or mild DR images may have more homogenous regions with low local entropy values.

Based on the training of the CNN by the entropy image of the gray level as shown in [18], the green component, UM, and bichannel CNN model are incorporated in this study to improve the detection performance and may assist ophthalmologists in evaluating retinal images for more accurate diagnoses.

## 4. Conclusions

A deep learning system can increase the accuracy for detecting or diagnosing retinal pathologies in patients with diabetes. The methodology of the proposed method first includes the green component of the RGB image. The entropy image of the green component can improve the accuracy and the sensitivity. Preprocessing by UM can provide better detection accuracy, sensitivity, and specificity. The bichannel CNN with the inputs of both the entropy images of the gray level and the green component preprocessed by UM further advances the detection of referable DR. The proposed deep learning technology can assist ophthalmologists in referable DR diagnosis and will be beneficial to the automated retinal image analysis system.

## Figures and Tables

**Table 1:** Performance comparison of various input images of the CNN for referable DR.

Input images of the CNN	Accuracy (%)	Sensitivity (%)	Specificity (%)
Original photograph [18]	81.80	68.36	89.87
Entropy image of the gray level [18]	86.10	73.24	93.81
Entropy image of the green component	87.27	76.70	93.07
Bichannel, entropy images (gray and green)	87.37	76.93	93.57
Entropy images of unsharp masking gray level	86.87	75.09	93.86
Entropy image of unsharp masking green component	87.41	76.75	93.28
Bichannel, entropy images (unsharp masking gray level and green component)	87.83	77.81	93.88

## Data Availability

The data used to support the findings of this study are available from the corresponding author upon request.
